# Emetic Tartar-Loaded Liposomes as a New Strategy for Leishmaniasis Treatment

**DOI:** 10.3390/pharmaceutics15030904

**Published:** 2023-03-10

**Authors:** Larissa D. Coelho, Mirna M. D. Souza, Geovanni D. Cassali, Raphaela A. Silva, Maria J. N. Paiva, André L. B. Barros, Eliane M. Teixeira, Josianne N. Silveira, Paulo M. Z. Coelho, Marta M. G. Aguiar, Mônica C. Oliveira

**Affiliations:** 1 Department of Pharmaceutical Products, Faculty of Pharmacy, Universidade Federal de Minas Gerais, Belo Horizonte 31270-901, MG, Brazil; 2 Department of Clinical and Toxicological Analysis, Faculty of Pharmacy, Universidade Federal de Minas Gerais, Belo Horizonte 31270-901, MG, Brazil; 3 Department of General Pathology, Institute of Biological Sciences, Universidade Federal de Minas Gerais, Belo Horizonte 31270-901, MG, Brazil; 4 Clinical Research and Public Policy Group on Infectious and Parasitic Diseases, René Rachou Institute, Fundação Oswaldo Cruz—FIOCRUZ, Belo Horizonte 30190-009, MG, Brazil; 5 Rene Rachou Institute, Oswaldo Cruz Foundation (FIOCRUZ), Belo Horizonte 30190-009, MG, Brazil

**Keywords:** liposomes, emetic tartar, visceral leishmaniasis, acute toxicity, antileishmanial efficacy

## Abstract

Emetic tartar (ET), was used in the treatment of leishmaniasis but its use was discontinued due to its low therapeutic index. Liposomes have been shown to be a promising strategy for delivery of bioactive substances in the region of interest, in order to reduce and/or eliminate undesirable effects. In the present study, liposomes containing ET were prepared and characterized to evaluate acute toxicity as well as their leishmanicidal action using BALB/c mice with an inoculum of *Leishmania* (*Leishmania*) *infantum.* Liposomes were composed of egg phosphatidylcholine and 3ß-[N-(N′,N′-dimethylaminoethane)-carbamoyl]cholesterol, with an average diameter of 200 nm, zeta potential of +18 mV, and ET encapsulated into liposomes at a concentration near 2 g/L. Healthy mice were treated with ET or liposome containing ET (Lip-ET) in a single dose of 16 mg/kg of Sb^3+^ intravenously and observed for 14 days. The death of two animals in the ET-treated group and no deaths in the Lip-ET-treated group was observed. Higher hepatic and cardiac toxicity were observed in animals treated with ET when compared to animals treated with Lip-ET, blank liposomes (Blank-Lip) and PBS. The study of antileishmanial efficacy was conducted by intraperitoneal administration of Lip-ET, for ten consecutive days. It was observed by limiting dilution that treatments with liposomal formulations containing ET, as well as Glucantime^®^, led to a significant reduction in parasitic load in spleen and liver (*p* < 0.05) when compared to the untreated control group.

## 1. Introduction

Leishmaniasis represents a group of diseases with a great clinical and epidemiological diversity, classified in the following main forms: cutaneous, visceral or kala-azar, and mucocutaneous leishmaniasis [1]. Visceral leishmaniasis (VL), also known as kala-azar, is fatal in more than 95% of patients when untreated. This disease is widespread in tropical and subtropical areas and is found in 98 countries in Europe, Africa, Asia, and America [2]. However, more than 95% of new cases of leishmaniasis occur in just ten countries, namely Brazil, China, Ethiopia, India, Iraq, Kenya, Nepal, Somalia, South Sudan, and Sudan. The symptoms of VL are irregular bouts of fever, weight loss, enlarged spleen and liver, and anemia [3].

The medicinal use of antimony compounds began in the 16th and 17th centuries with potassium antimony tartrate (emetic tartar, ET) for the treatment of lung diseases (pneumonia), typhoid fever, and schistosomiasis [4]. Anti-cancer activities of antimony agents have also been reported. Antimony tartrate has been found to be cytotoxic in vitro to various lung cancer cell lines. It is also reported that sodium stibogluconate is effective in suppressing hepatitis C virus replication [5]. Gaspar Vianna, a pioneering researcher of the study of Chagas disease and leishmaniasis, reported the efficacy of ET on treatment of mucocutaneous leishmaniasis. However, the clinical use of this compound has been discontinued because of its severe adverse effects such as gastrointestinal intolerance and cardiotoxic effects as well as due to discovery of new, less toxic drugs [6]. Pentavalent antimony-based compounds continue to be used as first choice treatment of all forms of leishmaniasis in humans [5]. Lipid formulations of amphotericin B are now competitive with pentavalent antimony as primary therapy for VL. Pentamidine, paromomycin, and adjuvant therapy with interferon-γ are secondary regimens for the treatment of this condition. Amphotericin B, pentamidine, and paromycin, when administered for long periods of treatment and higher doses, can cause several adverse effects (fever, reduced kidney function, myalgia), which are not so common for long periods with the use of antimonials [1]. In Brazil, the drug used is Glucantime^®^, which has severe adverse effects and is administered parenterally for 20 to 40 days, which compromises patient adherence to the treatment [6]. However, the mechanism of action of antimonials is still not fully understood. It is suggested that pentavalent antimony (Sb^5+^) is a prodrug, which is converted into trivalent antimony (Sb^3+^) after administration. However, the mechanism (enzymatic or non-enzymatic) and the site of parasite reduction have not yet been determined [7,8,9]. Sereno and coworkers (1998) demonstrated that Sb^3+^ was substantially more potent than Sb^5+^ for the promastigotes and axenic amastigotes of *L.* (*L.*) *mexicana*, *L.* (*L.*) *amazonensis*, and *L.* (*L.*) *infantum*.

Given these limitations, the World Health Organization (WHO) recommends research for new drugs and formulations as well as simpler and safer administration routes, such as oral and topical routes [10]. The development of a new drug is a time-consuming and expensive process. Therefore, the use of a pharmacologically known drugs and the development of new formulations have become interesting strategies, therefore, the idea of using liposomes containing ET is a viable one.

Liposomes are small, spherical-shaped artificial vesicles that can be created from natural and/or synthetic and non-toxic lipids. Liposomes are promising nanosystems as drug carriers because they are biodegradable and have an amphiphilic character [11]. Studies have shown that liposomes were able to reduce the acute toxicity of ET and effectively target the drug to *S. mansoni* during the course of infection [6].

The encapsulation of bioactive substances in liposomes may contribute to an increase in the bioavailability and fraction of bioactive substances reaching the pathological area, thereby improving therapeutic efficacy and minimizing toxicity [12].

The goals of this study were to prepare and characterize liposomes containing ET to evaluate their storage stability, acute toxicity in healthy mice, and initial efficacy in the treatment of the acute phase of experimental visceral leishmaniasis caused by *Leishmania* (*Leishmania*) *infantum* in BALB/c mice. The results of this study can serve as a base for future studies in the case of more extended research on this new potent alternative treatment for leishmaniasis.

## 2. Materials and Methods

### 2.1. Materials

Potassium antimony tartrate trihydrate (emetic tartar—ET) was obtained from Sigma-Aldrich (Saint Louis, MO, USA); Egg phosphatidylcholine (EPC) was obtained from Lipoid GmbH (Ludwigshafen, Germany); 3ß-[N-(N′,N′-dimethylaminoethane)carbamoyl] cholesterol (DC-CHOL) was obtained from Chem-Impex International, Inc. (Wood Dale, IL, USA); chloroform, phosphate anhydrous monobasic potassium, and HEPES (Vetec Quimica Fina Ltd.a, Duque de Caxias, Brazil); dibasic sodium phosphate heptahydrate (MERCK, Darmstadt, Germany); ethylenediamine tetraacetic acid (EDTA) (ECIBRA, São Paulo, Brazil); sodium chloride (MERCK, Darmstadt, Germany). The polycarbonate membrane was purchased from Millipore (Billerica, MS, USA). All reagents used were of analytical grade for Graphite Furnace Atomic Absorption Spectrometry (GFAAS), nitric acid at 65% (*v*/*v*) (HNO_3_) (MERCK, Darmstadt, Germany); hydrogen peroxide 30% (*v*/*v*) (Labsynth, Diadema, Brazil) and standard solution of 1000 mg L^−1^ of antimony (Sb) (MERCK, Darmstadt, Germany). Kits for biochemical analysis were obtained from Labtest (Lagoa Santa, Brazil) and Bioclin (Belo Horizonte, Brazil).

### 2.2. Liposome Preparation

Liposomes were prepared using the reverse-phase evaporation method [11]. Briefly, chloroform aliquots of EPC and DC-CHOL (7:3 molar ratio, at 20 mM lipid concentration) were transferred to a flask and solvent was removed on a rotary evaporator at low pressure to prepare Lip-ET and blank liposomes. Lipids were redissolved in treated diethyl ether, and a solution containing ET (60 g/L) in phosphate buffered saline (PBS) was added. The mixture was placed on rotary evaporator and organic solvent was removed under reduced pressure. The liposomes obtained were calibrated by extrusion using polycarbonate membranes of 0.4 µm (10 cycles) and 0.2 µm (5 cycles) (Lipex Biomembranes Extruder, Model T001, Vancouver, BC, Canada). After the vesicle size calibration step, non-encapsulated ET was removed by ultracentrifugation (ultracentrifuge Optima^®^ L-80XP, Beckman Coulter, Brea, CA, USA) at 150,000× *g*, at 4 °C, for 120 min and the pellet was resuspended with phosphate buffered saline (PBS buffer) pH 7.4.

### 2.3. Physicochemical Characterization of Liposomes

#### 2.3.1. Size, Polydispersity Index (PDI), Zeta Potential, and Morphology

Dynamic light scattering (DLS) was used to determine size and PDI of liposomes at 25 °C and 90° angles. Electrophoretic mobility associated with DLS was used to determine the zeta potential. The Zetasizer NanoZS90 (Malvern Instruments, Malvern, England) was used to perform the measurements. All samples were diluted in PBS buffer pH 7.4 in a ratio of 5:100 and the measurements were performed in triplicate [13].

Samples of liposome were imaged by cryogenic transmission electron microscopy (CRYOTEM). To prepare the samples a carbon film-coated grid was used and for negative staining was added 1% sodium phosphotungstate [14]. The images of the samples were obtained using a Tecnai G2-12—FEI Spirit Biotwin 120 kV transmission electron microscope at the Center of Microscopy at UFMG.

#### 2.3.2. Amount of Encapsulation

A method previously validated in the toxicology laboratory was used to quantify the percentage of encapsulation. ET quantification was performed by atomic absorption spectrometry using GFAAS (Varian SpectraA Zeeman, Yukon, OK, USA). Briefly, samples before and after ultracentrifugation were taken and diluted to the theoretical final concentration of 50 µg/L in water containing 0.5% (*v*/*v*) HNO_3_ and Sb was determined using pyrolysis and atomization temperatures at 700 °C and 2000 °C, respectively. The amount of antimony encapsulation (EP) was calculated according to the following equation:

EP(g/L) = [Sb] purified liposomes/[Sb] non-purified liposomes.

### 2.4. Preliminary Stability Assessment of Lip-ET

The storage stability of Lip-ET was evaluated at 7 and 15 days after their preparation. These formulations were maintained at 4 °C in a nitrogen atmosphere (*n* = 3). After each interval time, the samples were evaluated to determine diameter, polydispersity index, zeta potential, and ET content in the liposomes.

### 2.5. Acute Toxicity

In the acute toxicity study, healthy female BALB/c mice (20–25 g), 7 to 8 weeks old, were divided into four groups (*n* = 3 or 6 per group). Different single doses of ET and Lip-ET (32, 24, 20, 18, and 16 mg/kg of Sb^3+^) were administered intravenously (IV), and control groups received PBS or Blank-Lip (the dose of lipid was equivalent to 16 mg/kg Lip-ET). This study was approved by the Ethics Committee for Animal Experimentation of the Federal University of Minas Gerais (CEUA/UFMG) under protocol n^o^ 240/2018. After treatments, animals were observed for 14 days and behavioral/clinical modifications, body weight, morbidity, and mortality were evaluated [15]. To estimate the maximum tolerated dose (MTD), a baseline threshold was used at which all animals survived and during the experiment the bodyweight loss was less than 15% [13]. Animals were anesthetized with a mixture of ketamine (100 mg/kg) and xylazine (15 mg/kg) after 14 days and blood was collected in tubes containing anticoagulant (EDTA). Hematological parameters such as white blood cells (WBC), red blood cells (RBC), hemoglobin, lymphocytes and granulocytes were evaluated for each group. The parameters were measured on a hematology analyzer Hemovet 2300 (Sinothinker Tchnology, Shenzhen, China).

For biochemical analysis, blood was centrifuged (3500 rpm, 10 min) and plasma obtained was frozen at −20 °C. The tests were performed in the Bioplus BIO-2000 semiautomatic analyzer (São Paulo, Brazil) using commercial kits. Liver function was evaluated by determination of alanine aminotransferase (ALT) and aspartate aminotransferase (AST) activity; renal function was evaluated by the measurement of urea and creatinine; and cardiac injury, by dosage of creatine kinase—MB (CK-MB) activity.

Liver, kidneys, spleen, and heart were collected for histopathological analysis. Buffered formalin (10%) was used to fix the samples followed by dehydration in alcohol and embedding in paraffin blocks. After obtaining 4 µm sections, they were stained with hematoxylin and eosin (H&E) [13]. A trained pathologist evaluated the sections and to capture images a camera connected to an optical microscope (Olympus BX-40; Olympus, Tokyo, Japan) was used.

### 2.6. In Vivo Antileishmanial Efficacy

Healthy female BALB/c mice (20–22 g) were inoculated intravenously with 0.1 mL volume of 2 × 10^7^ stationary growth phase promastigotes of *L.* (*L.*) *infatum* (MHOM/BR/74/PP75) as previously described by Santos and Col (2018) [16]. This study was approved by the Ethics Committee for Animal Experimentation of the Federal University of Minas Gerais CEUA/UFMG) under protocol n^o^ 128/2016.

Treatment was initiated 7 days after infection. Animals were divided into four groups (*n* = 7 per group): untreated control group; Blank-Lip (the concentration of lipids administered was the same used in the administration of Lip-ET at a dose of 7.8 mg/kg); Lip-ET (7.8 mg/kg/day of Sb^3+^); ET (7.8 mg/kg/day of Sb^3+^) and Glucantime^®^ (150 mg/kg/day of Sb^5+^). Mice were treated for 10 consecutive days intraperitoneally (IP)—0.2 mL.

The effectiveness of treatments was evaluated by the determination of viable parasites in spleen and liver using limiting-dilution assay, as previously described [16]. Briefly, the spleen and the liver were immediately removed, weighed, and homogenized with an Ultra-Turrax^®^ (IKA, Staufen, Germany), in Schneider’s medium containing 100 U/mL penicillin and 100 µg/mL streptomycin solution. The homogenate was submitted to serial dilutions in triplicate (1:10) in sterile 96-well culture plates and incubated at 23 °C. Each well was examined for the presence of parasites and the number of parasites was determined by the highest dilution at which parasites could grow over a 7 day period. The lowest dilution at which parasites could be detected was 10^1^, which was considered to be the limit of quantification, and the highest was 10^11^.

The toxicity of treatment was evaluated by determination of weight of animals on the first and last day of treatment. Other signs such as piloerection and survival were observed as indicators of systemic toxicity.

### 2.7. Statistical Analysis

Acute toxicity study data were expressed as the mean ± standard deviation (SD). The normality and homoscedasticity of variables were verified by Shapiro–Wilk and Brown–Forsythe, respectively. Then, data were evaluated by analysis of variance (ANOVA), and if statistical difference was observed, results were submitted to Tukey’s test. Differences were considered significant for *p* values less than 0.05. The data obtained for parasitic burden in liver and spleen were transformed into log (*N*+1) and submitted to the Kolmogovov–Smirnov (KS) normality test and Bartlett’s homoscedasticity test. Then, data were evaluated by analysis of variance (ANOVA), and if statistical difference was observed, results were submitted to Tukey’s test. Differences were considered significant for *p* values less than 0.05. The graphics and statistical analyses were performed using GraphPad Prism^®^ software.

## 3. Results

### 3.1. Physicochemical Characterization of Liposomes

**Figure 1:**
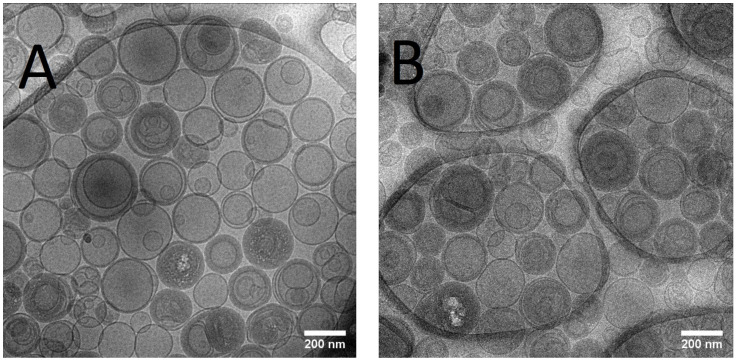
(**A**,**B**) CRYOTEM images of liposomes containing ET extruded through a 400 nm and 200 nm filter, respectively.

The mean size of Lip-ET and Blank-Lip values were equal to 230 nm and 163 nm, respectively (Table 1). The PDI values were less than 0.3, indicating the formation of monodisperse systems. The positive values of zeta potential for Lip-ET (17.3 ± 1.3 mV) and Blank-Lip (21.6 ± 1.2 mV) were due to the presence of the cationic lipid 3ß-[N-(N′, N′-dimethylaminoethane)-carbamoyl]cholesterol. The amount of ET encapsulated in these cationic liposomes was equal to 2.34 ± 0.32 g/L. Figure 1 shows CryoTEM images of Lip-ET extruded through a 400 nm filter (ten times) and a 200 nm filter (five times). The median size was 200 nm, confirming the results obtained by DLS. Most vesicles were unilamellar; also observed was the presence of some vesicles containing smaller ones inside of them.

### 3.2. Preliminary Stability Assessment of Lip-ET

**Figure 2:**
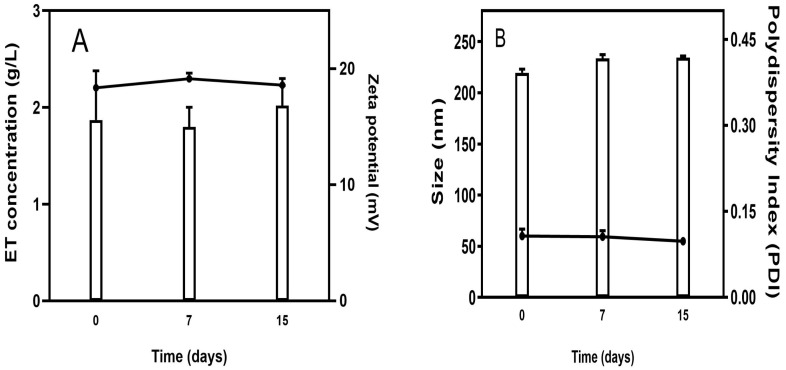
(**A**) Evaluation of the concentration of ET retained in liposomes and zeta potential during storage; (**B**) Evaluation of the average size and PDI of Lip-ET. Data are expressed as the mean ± standard deviation of the mean (*n* = 3).

The stability study showed that ET concentration was not changed over 15 days of storage, indicating excellent stability of the Lip-ET formulation (Figure 2A). Similarly, no significant variations (*p* > 0.05) were observed in relation to the zeta potential values. Regarding the size of vesicles, no variation was observed, and the values remained near 200 nm. The value of PDI remained constant over 15 days, and below 0.3, indicating that the Lip-ET formulation remained monodisperse (Figure 2B).

### 3.3. Acute Toxicity

All mice that received Lip-ET or ET treatments at dose of 32, 24, 20, and 18 mg/kg of Sb^3+^ (*n* = 3 per group) died, except in the case of Lip-ET treatment at dose 20 mg/kg of Sb^3+^ (two mice died).

**Figure 3:**
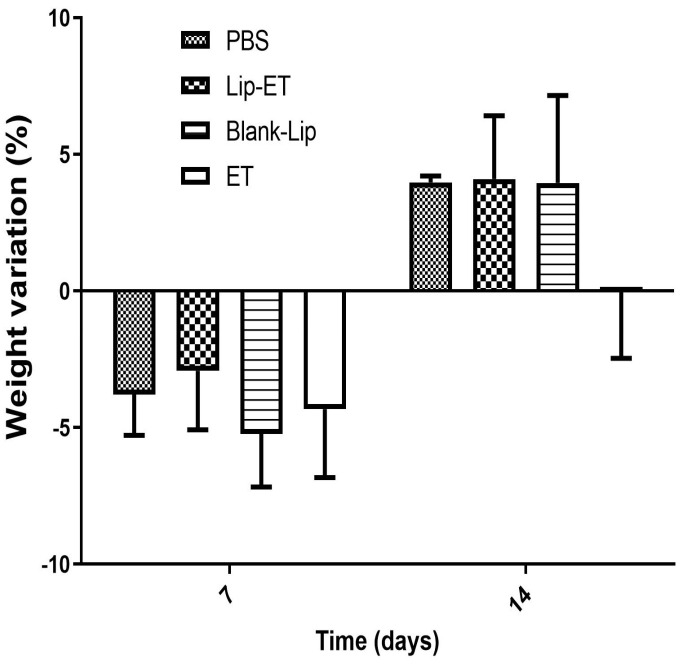
Female BALB/c mice body weight variation percentage after 7 and 14 days of intravenous administration of ET solution (16 mg Sb^3 +^/kg/day) (ET), Lip−ET (16 mg Sb^3 +^/kg/day), Blank−Lip and PBS (control) (*n* = 6 for each group). Data are the mean percentage of weight variation percentage ± standard deviation of the mean.

The body weight variation of mice after Lip-ET and ET treatments at a dose of 16 mg/kg of Sb^3+^ is shown in Figure 3. After the first 7 days, a loss of body weight was observed for mice submitted to all treatments, without any statistical differences. After 14 days, a body weight gain in mice was observed for PBS, Blank-Lip, and Lip-ET treatments. Mortality was observed only for mice treated with ET (two animals), while two animals also presented pronounced clinical toxicity signs, such as prostration and intense piloerection. All mice treated with Lip-ET and Blank-Lip presented discrete piloerection and prostration only during the first 2 h following administration. These findings indicate that the encapsulation of ET into liposomes can reduce the toxicity of ET.

Hematological and biochemical parameters of mice treated with PBS, Lip-ET, Blank-Lip, and ET are shown in Table 2. No significant difference was observed for the hematological parameters, such as red blood cells (RBC), hemoglobin, white blood cells (WBC), lymphocytes, and granulocytes.

**Figure 4:**
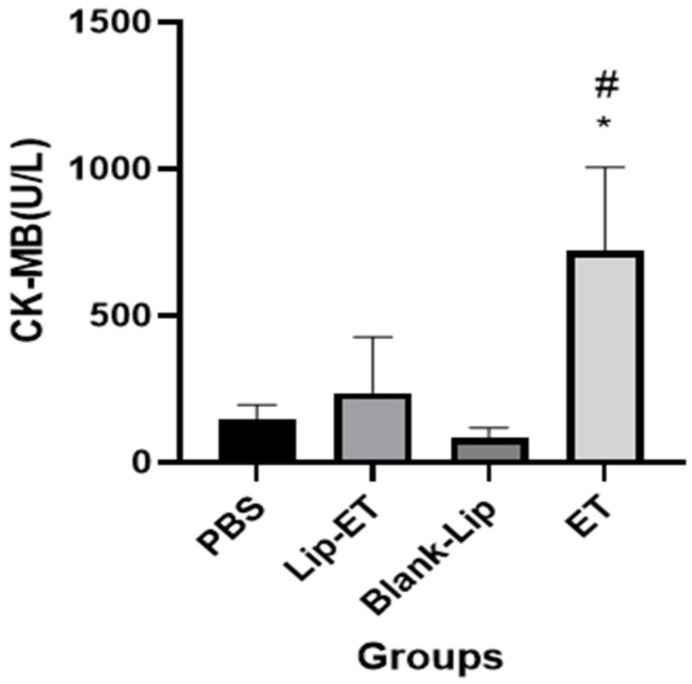
Evaluation of the CK-MB activity of BALB/c female mice 14 days after intravenous administration of PBS, Lip−ET (16 mg/kg of Sb^3+^), Blank−Lip and ET (16 mg/kg of Sb^3+^) (*n* = 6 for each group). Data are the mean ± standard deviation of the mean. # Indicates significant statistical difference (*p* < 0.05) between control and ET treated groups. * Indicates significant statistical difference (*p* < 0.05) between Lip-ET and ET treated groups. All data were analyzed by one-way ANOVA analysis of variance followed by Tukey’s test.

Biochemical parameters indicative of cardiac (CK-MB), hepatic (ALT and AST) and renal (urea and creatinine) toxicity were analyzed. A significant increase in CK-MB activity was observed for ET-treated animals (16 mg/kg of Sb^3+^), when compared to PBS, Lip-ET, and Blank-Lip treated groups (Figure 4).

**Figure 5:**
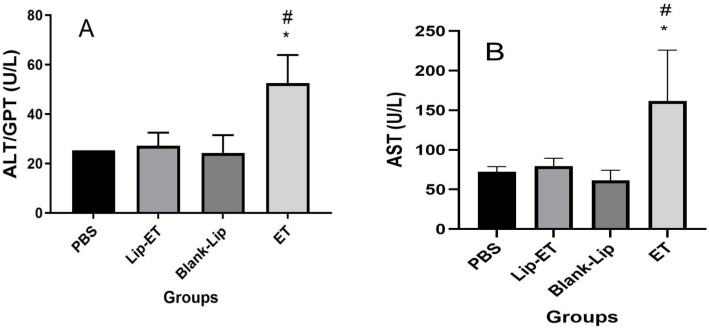
Evaluation of hepatic toxicity of BALB/c female mice 14 days after intravenous administration of PBS, Lip−ET (16 mg/kg of Sb^3+^), Blank−Lip and ET (16 mg/kg of Sb^3+^) (*n* = 6 for each group). (**A**,**B**) show ALT and AST activity, respectively. Data are the mean ± standard deviation of the mean. # Indicates significant statistical difference (*p* < 0.05) between control (PBS) and ET treated groups. * Indicates significant statistical differences (*p* < 0.05) between Lip−ET and ET treated groups. All data were analyzed by one-way ANOVA analysis of variance followed by Tukey’s test.

Concerning evaluation of hepatic toxicity, a significant increase in ALT and AST levels were observed for ET-treated animals (16 mg/kg of Sb^3+^) when compared to PBS, Lip-ET, and Blank-Lip treated groups (Figure 5A,B). However, no significant statistical difference was observed in levels of urea and creatinine between groups of treatment (Table 2).

**Figure 6:**
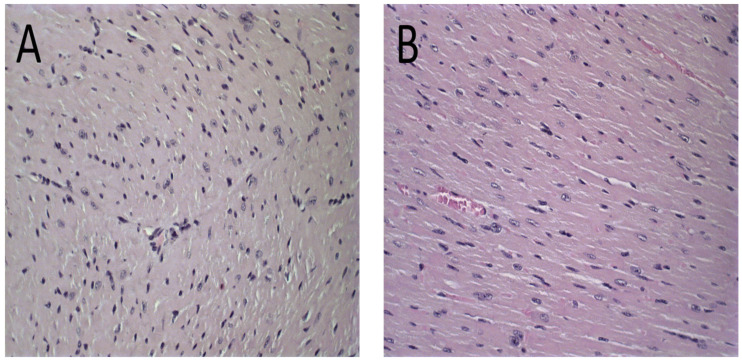

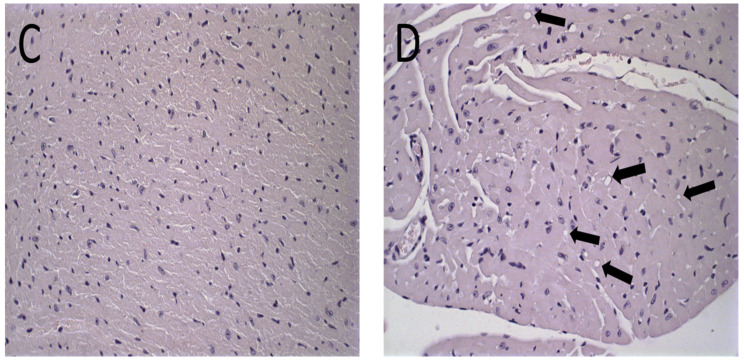
Histological sections of female BALB/c mice hearts stained by hematoxylin and eosin. (**A**) PBS, (**B**) Blank-Lip, (**C**) Lip-ET (16 mg/kg of Sb^3+^) and (**D**) ET (16 mg/kg of Sb^3+^). The black arrows indicate vacuoles in cardiomyocytes.

**Figure 7:**
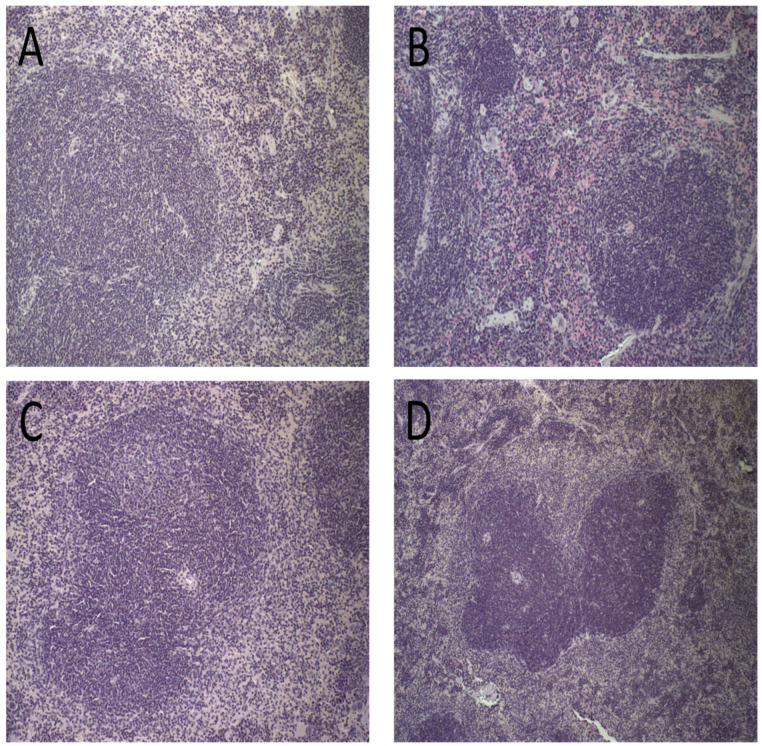
Histological sections of female BALB/c mice spleen stained by hematoxylin and eosin after treatment with PBS (**A**), Blank-Lip (**B**), Lip-ET (16 mg/kg of Sb^3+^) (**C**) and ET (16 mg/kg of Sb^3+^) (**D**).

The histological analysis revealed that mice treated with PBS, Blank-Lip, and Lip-ET presented the same histopathological profile. No changes were observed in the PBS group for all organs analyzed (Figure 6A and Figure 7A). For renal and hepatic histopathology, occasional tissue changes were observed, but with no significant difference between groups.

The histological analysis of the heart revealed that the mice that received ET treatment (16 mg/kg of Sb^3+^) presented a focal area of mild cardiomyocyte vacuolization, compatible with steatosis (Figure 6D). However, the mice treated with Lip-ET and Blank-Lip showed a histological profile of the heart similar to that of PBS-treated mice (Figure 6A–C).

Spleen photomicrographs are illustrated in Figure 7. Morphological changes were observed in all animals receiving ET (16 mg/kg of Sb^3+^), revealing a discreet red pulp hyperplasia (Figure 7D). By contrast, no alteration of the splenic tissue was observed for PBS, Blank-Lip, and Lip-ET treated groups (Figure 7A–C).

### 3.4. In Vivo Antileishmanial Efficacy

**Figure 8:**
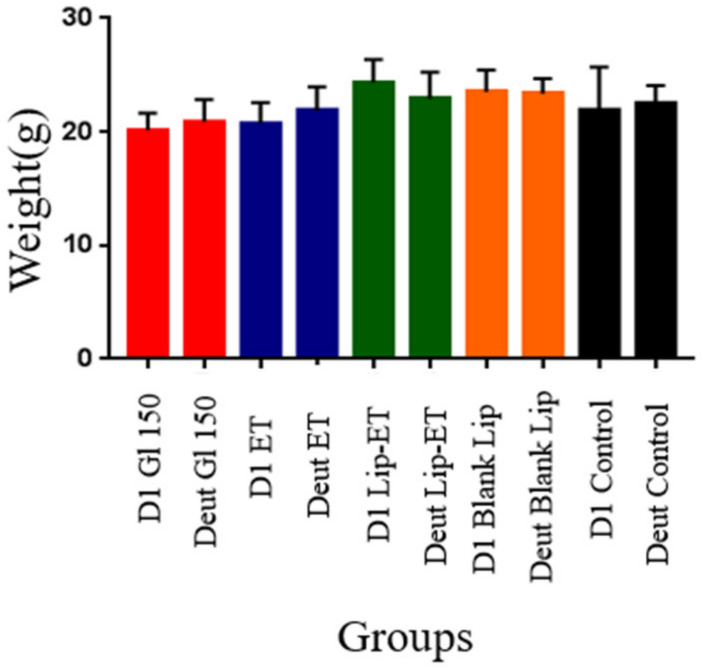
Weight of BALB/c mice infected with *L.* (*L.*) *infantum* and subjected to different treatments on day 1 (D1) and on the day of euthanasia (Deut). The treatments used were Glucantime^®^ 150 mg/kg/day (Glu 150), ET solution (7.8 mg Sb^3 +^/kg/day) (ET), liposome containing ET (7.8 mg Sb^3 +^/kg/day) (Lip-ET), blank liposomes (Blank-Lip) and untreated group (Control) (*n* = 7 for each group).

The effectiveness of treatments was evaluated experimentally in female BALB/c mice infected with *L.* (*L.*) *infantum*. Regarding the weight of animals on the first day of treatment and on the euthanasia day, there was no statistically significant difference between the groups of treatment (Figure 8) (*p* > 0.05). No other signs of systemic toxicity such as prostration or agitation, diarrhea, convulsions, piloerection or death were observed in all evaluated groups. It is worth emphasizing that this result may be due to the use of an ET dose twice as low as that used in the acute toxicity study.

**Figure 9:**
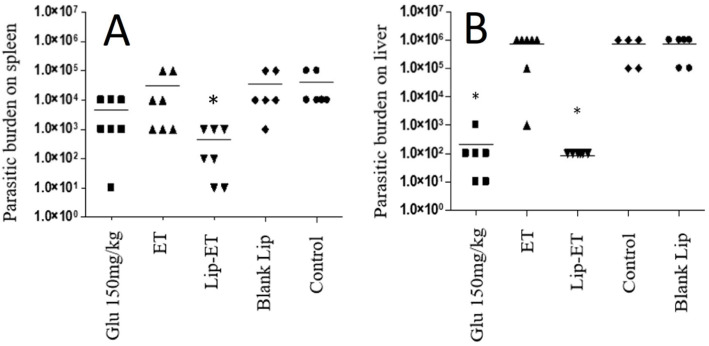
Evaluation of the parasitic burden in the spleen (**A**) and liver (**B**) of BALB/c mice infected with *L.* (*L.*) *infantum* and submitted to different treatments (*n* = 7 for each group). The treatments used were Glucantime^®^ 150 mg/kg/day (Glu 150 mg/kg), ET solution (7.8 mg of Sb^3 +^/kg/day) (ET), Lip-ET (7.8 mg of Sb^3 +^/kg/day), blank liposomes (Blank-Lip) and untreated group (Control). Animals were treated for ten consecutive days intraperitoneally. Three days after the end of treatment, the parasitic burden was determined by the limiting dilution method. * Indicate significant statistical differences (*p* < 0.05) between control and treated groups. Dashes indicate the mean and symbols the individual values. Data were analyzed by one-way ANOVA analysis of variance followed by Tukey’s test.

The parasitic burden on the spleen and liver was assessed 3 days after the end of treatment and results are shown in Figure 9A,B. Significant antileishmanial efficacy of Lip-ET treatment can be observed by reducing the parasite load in the liver and spleen (*p* < 0.05). This reduction in the liver was equivalent to that obtained with the use of conventional treatment with Glucantime^®^ and was even greater in the spleen compared to this treatment. Lip-ET showed 99.0% and 99.9% suppression of spleen and liver infection, respectively, while Glucantime^®^ was able to lead to 88.2% and 99.9% suppression in these organs. Interestingly, we observed that ET treatment, administered at the same dose for Lip-ET, was only able to lead to a slight reduction in infection (20.4% and 14.3%, in spleen and liver, respectively).

## 4. Discussion

Leishmaniasis presents an important clinical and epidemiological diversity and is caused by pathogens of the genus *Leishmania*. The disease can be asymptomatic in humans and in animal reservoirs. Furthermore, a single species of *Leishmania* can cause several forms of the disease [17].

Since the last half of the 20th century, the treatment of leishmaniasis has been with the use of pentavalent antimonials, but in the last two decades these have shown an increase in clinical resistance.

Among the factors implicated in antimony resistance are decreased biological reduction of Sb^5+^ to Sb^3+^ and increased levels of trypanothione, which provides increased thiol redox potential [18]. An approach that could circumvent these drawbacks and enable the use of antimonials for the treatment of leishmaniasis would be the use of a bioactive compound containing Sb^3+^, such as ET [13]. However, its low therapeutic and cardiotoxic effects prevent its use as a leishmanicidal drug [19]. In this context, the use of drug delivery systems can be a strategy to reduce the toxicity of ET and also contribute to a better leishmanicidal efficacy [18,20,21].

Nanotechnology and nanoscience present a highly positive prospective of bringing benefits to many research areas and applications. Nanosized vehicles have received considerable attention as drugs carriers [12]. Thus, Lip-ET is an innovative liposomal formulation presenting physicochemical characteristics suitable for intravenous administration, such as size (230.0 ± 7.8 nm) and PDI (0.134 ± 0.01) of the vesicles. These parameters are of fundamental importance in formulation of liposomes, being related not only to the stability but also to the safety of these systems in the parenteral administrations [12]. To improve treatment efficacy and reduce toxicity, an innovative liposomal formulation of trivalent antimony was developed.

Surface charge, vesicle size, and membrane fluidity are physicochemical properties that influence stability of liposomes. A typical phenomenon of instability upon storage is the increase in the particle size provoked by aggregation or fusion of unstable liposomes [22]. The preliminary stability of liposomes was observed in our study, since all evaluated chemical and physicochemical parameters remained unchanged over the time evaluated. This behavior may be due to the repulsion provoked by the presence of positive charge on their surface, resulting from the presence of the cationic lipid 3ß-[N-(N′,N′-dimethylaminoethane)-carbamoyl]cholesterol, which could prevent vesicle aggregation, ensuring the stability of the formulation [10].

The acute toxicity evaluation study is an important step when the objective is introduction of new pharmaceutical products for clinical use [23]. Currently, an increase in the study and use of nanoparticles is being observed. However, due to the lack of specific regulation for nanoparticles, studies involving the toxicity of these new pharmaceutical forms are still controversial in terms of standardization and results. However, some regulatory bodies, such as the FDA, recommend the study of toxicity for liposomal systems using the encapsulated form, the free drug and the pure carrier [24]. Regarding the hepatic toxicity, the protection against hepatic damage was observed when ET was encapsulated in liposomes composed of EPC and DC-CHOL (7:3 ratio, at 20 mM lipid concentration).

It is noteworthy that the use of cationic liposomes may have contributed to the absence of liver toxicity determined by the quantification of the liver damage biomarkers ALT and AST. It is well known in the literature that cationic liposomes remain longer in the bloodstream than neutral ones, and therefore cationic liposomes are taken up less by the liver [25]. The decreased hepatic uptake of Lip-ET may also explain the absence of inflammatory infiltrate and associated hyperemia when compared to that observed after administration of neutral liposomes composed of diestearoylphosphatidylcholine and cholesterol into *L. infantum* (C43 strain)-infected mice [26]. Although there may have been a reduction in the hepatic uptake of Lip-ET, its extension did not impair the leishmanicidal efficacy. The reduction in the parasitic load was very significant, reaching a level similar to that obtained with the administration of Glucantime^®^ at a dose 19 times higher. The spleen is another organ of the mononuclear phagocytic system involved in the uptake of nanosystems; however, there was no injury to this tissue after the administration of Lip-ET. In addition, Lip-ET was able to induce a greater reduction in splenic parasite load compared to other treatments, including Glucantime^®^.

Cardiotoxicity is the most well-known, serious, and debilitating adverse effect after administration of ET, being characterized by changes in the electrocardiogram, inversion of the ST segment, prolongation of the QT interval, and consequently, the appearance of arrhythmias and sudden cardiac arrest [19]. In the present study, an increase in the serum levels of CK-MB (isoenzyme released into the circulation in cases of cardiac injury) was observed in animals treated with ET when compared to the other treatment groups. The encapsulation of ET in cationic liposomes also allowed the elimination of cardiotoxicity induced by ET treatment. In addition, the administration of Lip-ET did not induce active hyperemia throughout the myocardium, which was observed in the case of injection of liposomes composed of diestearoylphosphatidylcholine and cholesterol containing ET [26]. Similar behavior was observed for these cationic and neutral liposomes in relation to renal toxicity.

Supported by our toxicological results for Lip-ET, we have established that such a formulation, administered at 7.8 mg of Sb^3+^/kg/day, could represent a safe and effective dose. In fact, after treatment, the animals submitted to the treatment did not show weight loss or signs of toxicity and only Lip-ET showed a reduction in the parasitic load in all organs investigated. The parallel administration of free Sb^3+^(ET solution) at the same dose, did not show antileishmanial efficacy. Such findings confirmed the ability of liposomes to direct the active compound to tissues containing cells of the mononuclear phagocytic system (MFS) [26,27]. Other studies have also shown administration of antimonial drugs using conventional liposomes to be effective compared to non-encapsulated antimonials in experimental VL [28,29]. On the other hand, treatment with Glucantime^®^, a reference drug in the treatment of VL, was effective in reducing the parasite load in the liver, which can be explained by the higher levels of antimony uptake in the liver compared to the spleen of BALB/c infected mice [30,31]. In addition, studies with liposomes were evaluated in an experimental model of schistosomiasis; mice received ET intraperitoneally, either in free form or in encapsulated form. Only the group treated with liposomes showed a significant reduction in the parasite load, and when subjected to a higher dose of antimony, all mice that received ET in free form died, while those that received the form encapsulated in liposomes survived [6].

It is important to emphasize that the effectiveness of the liposomal delivery system depends on its capture by the MFS cells. This process can be facilitated by the opsonization of liposomes, as well as by the route of administration [32]. Indeed, when intraperitoneally administered drugs are exposed to the large surface area of the peritoneal membrane, rapid absorption is expected. In our study, as the established treatment protocol was for ten consecutive days, we opted for the intraperitoneal route of administration over the intravenous route, as the latter could be painful for the animals for such a long period. It is expected that the pharmacological and/or toxic effects caused by substances injected intraperitoneally and intravenously are very close [33].

## 5. Conclusions

The results pointed to the safety and *antileishmanial* efficacy of the Lip-ET formulation in reducing the parasite load in the spleen and liver of animals experimentally infected with *L.* (*L.*) *infantum*, thus being an interesting alternative for the treatment of VL.

## Figures and Tables

**Table 1:** Chemical and physico-chemical characterization of Lip-ET and Blank-Lip.

Groups	Size (nm)	Polydispersity Index (PDI)	Zeta Potential (mV)	Encapsulation Content (g/L)
Lip-ET	230.0 ± 7.8	0.134 ± 0.01	+17.3 ± 1.3	2.34 ± 0.32
Blank-Lip	163.0 ± 7.7	0.076 ± 0.01	+21.6 ± 1.2	-

Data are expressed by the mean (*n* = 3) ± standard deviation.

**Table 2:** Hematological and biochemical parameters of female BALB/c mice treated with PBS, Lip-ET, Blank-Lip and ET.

Variable	PBS	Lip-ET (16 mg/kg Sb^3+^)	Blank-Lip	ET (16 mg/kg Sb^3+^)
RBC (10^6^/µL)	6.58 ± 0.08	6.69 ± 0.65	6.63 ± 0.12	6.82 ± 0.83
Hemoglobin (g/dL)	12.60 ± 0.26	13.12 ± 1.68	12.93 ± 0.35	14.00 ± 3.62
WBC (10^3^/µL)	4.17 ± 0.57	5.42 ± 1.41	5.27 ± 0.80	4.37 ± 1.40
Lymphocytes (10^3^/µL)	1.7 ± 0.5	1.8 ± 0.8	1.8 ± 0.3	1.7 ± 0.6
Granulocytes (%)	53.6 ± 18.36	42.48 ± 10.02	43.93 ± 1.83	31.35 ± 12.37
Creatinine (mg/dL)	0.32 ± 0.03	0.28 ± 0.07	0.36 ± 0.06	0.42 ± 0.21
Urea (mg/dL)	41.32 ± 2.23	35.37 ± 9.74	39.62 ± 1.11	28.44 ± 6.41

RBC and WBC means red blood cells and white blood cells, respectively. Data were expressed as mean ± standard deviation of the mean (*n* = 6 for each group).

## Data Availability

Data available in a publicly accessible repository that does not issue DOIs. Publicly available datasets were analyzed in this study. This data can be found here: http://hdl.handle.net/1843/40508.
